# Death from Lodgment of a Gold Plate in the Bronchial Tubes

**Published:** 1886-04

**Authors:** J. W. Jay

**Affiliations:** Richmond, Ind.


### Death from Lodgment of a Gold Plate in the Bronchial Tubes.

BY DR. J. W. JAY, RICHMOND, IND.

Read at the Forty-Second Annual Meeting of the Mississ'ppi Valley Association.

ENOS MOTE, the subject of this writing, resided near West Milton, Miami County, Ohio, sixteen miles north of Dayton. About the 1848 he had his four upper central teeth knocked out by a handspike, while trying to roll a heavy log. In the course of time he had those teeth artificially substituted by Dr. Sylvanus Tolbert, who was then a leading dentist of the city of Dayton.

He had been afflicted occasionally with something like epilepsy, and in the evening on the third Sabbath in August, 1855, he had a fit on the public highway, near his father's house, into which he was carried, and where he remained during the night. Near 3 o'clock in the morning, he was seized with another one. His wife called for assistance, he struggled fearfully; and it was soon perceived that his teeth were gone. After the paroxysm had ceased, and daylight had dawned, diligent and persistent search was made for them, both in and out of the house, but to no purpose. His wife said she recollected pushing them into place, after he had recovered from the spasm on the evening before, one side having dropped down from its position. During the day following the loss of the teeth, he had frequent and severe paroxysms of coughing, and also during the succeeding night, accompanied with severe pain, and a feeling as though there were a lump of something in his left lung as large as his fist. As nearly as he could tell, from the sensation that he had, it was located at the region of the bifurcation of the bronchial tubes, from the main trunk. Within a few days a hemorrhage, more or less severe, followed each paroxysm of coughing, attended with intense pain. A nephew of his, with whom I am well acquainted, and who has kindly furnished me with the facts in the case, though but a lad at the time, fully believed that the teeth were in the lung of his uncle, and were producing all this disturbance. He gave it his opinion that the teeth had gone into the lung during his struggles in the fit that he had at 3 o'clock in the morning. He so stated his convictions to the attending physicians, who entirely discarded the idea, and gave it as their opinion that it would have been impossible for him to have lived five minutes with such a foreign substance in his lung.

The case went on with no satisfactory solution in reference to the loss of the teeth, and the cause of the physical condition under which he was laboring. He was faithfully attended by his family physician and others in consultation, who administered to his needs according to the best of their ability, without being able to afford him any permanent relief. As might be expected, under the circumstances, his sufferings were very great, and finally he began to expectorate very freely, and gradually grew worse, from week to week, and was held very much the same as one going down with phthisis pulmonalis. In fact the doctors said it was that. Finally he died in August, 1856, five days less than one year from the time of the disappearance of the teeth. This nephew, who was with him the most of the time during his decline, suggested and urged a *post mortem* examination, but the father of the dead man, being very old, objected, saying that he could not bear the idea of it, and the physicians did not think it worth while, as he had undoubtedly died of consumption. And being only a lad, without the necessary influence, he was forced to give it up, but without being convinced that he was wrong in his conclusions. Twelve years after the death of his uncle, he was in Iowa, and in the office of a physician, a cousin of his, he picked up a medical journal, and in looking over its pages, he came across the report of a case, in which a man had gotton a foreign substance in one of his lungs, which resulted in death. The attending symptoms detailed in this case, corresponded so exactly to those of his uncle's, that he was more than ever convinced that he was right in his conclusions in regard to the loss of teeth, and the immediate cause of his uncle's sickness and death. He therefore determined, on returning home, to go down into the grave and forever settle the question, which he did accordingly along with others, in April, 1868. They found the coffin pretty well decayed, but the skeleton in a good state of preservation, and the teeth intact, just as you see them, resting between the fifth and sixth ribs. Months before his death, the exhaltations from his body, his breath, and the expectorated matter, were so exceedingly offensive that it was almost impossible for any one to remain long in the room with him.

